# Experimental Study of Maintenance-Free Steel-Composite Buckling Restrained Braces

**DOI:** 10.3390/ma15165538

**Published:** 2022-08-11

**Authors:** Jun Zhao, Huiping Wang, Jun Dong, Liwei Zhang

**Affiliations:** 1 School of Management Engineering, Jiangsu Urban and Rural Construction Vocational College, Changzhou 213000, China; 2 College of Civil Engineering, Nanjing Tech University, Nanjing 211816, China

**Keywords:** maintenance-free brace, steel-composite structure, buckling restrained brace, quasi-static test, hysteretic behavior

## Abstract

High-rise buildings are very flexible, have a small damping, and respond significantly under strong dynamic load. However, there is a lack of studies about buckling restrained braces (BRBs) used in high-rise buildings, towering steel structures, and bridge structures. In this paper, the authors put forward a new type of brace, the maintenance-free steel-composite buckling restrained brace (MFSC-BRB). The MFSC-BRB has a steel plate core, for which a ribbed glass fiber reinforced polymer (GFRP) rectangular tube is used as the restraint unit; the steel core is encapsulated by the GFRP rectangular tube. In this study, MFSC-BRBs were fabricated using a vacuum-infused molding process that enabled them to be integrally formed all at once. Four specimens were designed according to the vacuum-infused molding process; then, the failure mode, deformation capacity, and hysteretic behavior of the MFSC-BRBs were studied. The results showed that the new MFSC-BRB has good integrity and good energy dissipation capacity under the action of a low weekly repeated loading effect. When the axial displacement is large, the performance of the single wave section expansion joint is better than that of the double wave section expansion joint. The greater the restraint ratio of the restrained yield section, the greater the energy dissipation of the brace. The proposed MFSC-BRBs have good integrity and are lightweight compared with traditional BRBs.

## 1. Introduction

Buckling restrained braces (BRBs) are a new type of brace which have been studied extensively in recent years. They are generally composed of a core unit and a peripheral restraint unit. The core unit mainly bears axial load, while the peripheral restraint unit mainly increases the anti-lateral stiffness of the brace to ensure that the core unit bears most of the axial force without destabilization. They are an economical and efficient energy-dissipating brace because of their convenient fabrication. However, BRBs are seldom used in high-rise buildings. The application of BRBs to high-rise buildings can effectively control the displacement, and can achieve a good vibration reduction effect. The application of BRBs to the vibration reduction design of bridge structures can provide a new way to solve the vibration reduction design of high-rise buildings under rare earthquakes [1,2,3,4,5].

The form and performance of restrained parts play a decisive role in the overall structural performance of BRBs, and the research and development of BRBs also reflects the characteristics of restrained parts form innovation as the main consideration. BRBs are divided into the overall constraint type and assembly constraint type according to the constraint form.

Integral restrained BRBs can be divided into reinforced concrete restrained braces, steel tube composite restrained braces, and steel restrained braces according to the main materials. Mochizuki et al. [6] and Nagao et al. [7] studied the performance of two common BRBs restrained by reinforced concrete.

In order to solve the problem of the rapid attenuation of peripheral restrained stiffness due to concrete cracking in BRBs of reinforced concrete, Kimura et al. [8] first carried out research on concrete-filled steel tubular restrained BRBs. Peripheral steel pipes bound the filled concrete and at the same time served as a formwork for the concrete placement, significantly reducing the processing complexity and cost. Guo Y L et al. [9] carried out in-depth research on the influence of the restraining rigidity, the width-to-thickness ratio of the core plate, and the initial defects of CFST BRBs on the overall mechanical behavior of members.

A BRB restrained by pure steel can effectively avoid the construction difficulty caused by concrete placing and maintenance, and can also show improved construction accuracy. At the same time, the weight of the BRB members is significantly reduced, which makes the structural arrangement more flexible. It can also achieve better non-bonding performance by precisely controlling the gap size between the inner core and outer restrained members, without bonding the non-bonding material. Suzuki et al. [10], Shimizu et al. [11], and Usami et al. [12] carried out theoretical and experimental research on several common types of all-steel integrally restrained BRBs. Zhou Y et al. [13,14] proposed a triple buckling brace of steel pipes formed by double-layer steel pipes with an inner core pipe clamped in the middle, and further developed the triple buckling brace of steel pipes with an inner core opening/groove, which can control the inner core yielding only in the area of the opening/groove section, thus reducing the risk of local failure of the inner core extension section of the buckling brace.

The peripheral restrained member of the assembled buckling brace consists of several components, which are joined as a whole by high-strength bolts. The advantage is that the bolt connection is convenient. Components with a large self-weight can be dismantled and transported separately to reduce the difficulty of transportation and installation. Due to the disassembly of peripheral restrained members, the damaged core can be easily replaced in post-earthquake structural repair to reduce the cost of structural repair. Since the assembly can be prefabricated and the relative position of the assembly can be flexibly adjusted by the thickness of bolt gaskets, the machining accuracy of the components can be significantly improved and the requirement of adding non-bonding material between the core and the periphery can be avoided. On the other hand, unlike the integral restrained type, the restrained components of the assembled BRB are connected by bolts with interval distribution. The peripheral restrained stiffness generated by this discrete connection method must be reduced to some extent on the basis of the integral section, which is also the focus of the performance study of the assembled BRB. According to the different materials of peripheral restrained components, the assembled buckling brace is divided into three types: reinforced concrete assembly, concrete filled steel tube assembly, and pure steel assembly.

The earliest RC assembled buckling brace was proposed by Inoue et al. [15]. Its peripheral restraining member consists of two prefabricated RC slabs connected by long and high-strength bolts. The prefabrication of reinforced concrete is more suitable for industrial production with controllable precision, but it still cannot solve the cracking of peripheral concrete under the action of the transverse compression force of the core.

Research on the assembled BRBs of concrete-filled steel tubes (CFSTs) is mainly carried out in Japan and Taiwan. The energy-dissipating core section was proposed by Chou et al. [16].

In order to make full use of the advantages of the convenient and lightweight connection of assembled BRBs, many new section types of pure steel assembled BRBs have been developed, such as the square steel pipe restrained type, double T restrained flat steel plate inner core type, and many new section types of all-steel assembled BRBs. Zhou Y et al. [17] systematically studied an assembled BRB with a perforated steel plate and established the analysis theory and design method.

In this paper, the basic structure of a new type of MFSC-BRB is proposed, and the working principle of the new MFSC-BRB is analyzed. Based on the vacuum-infused molding process, four new scale models of the MFSC-BRB are designed and fabricated. The performance indexes of the new MFSC-BRB, such as its integrity, energy dissipation capacity, ductility, and stiffness degradation under repeated low-cycle loading, are studied.

## 2. Theoretical Study

### 2.1. Basic Structure of the MFSC-BRB

**Figure 1:**
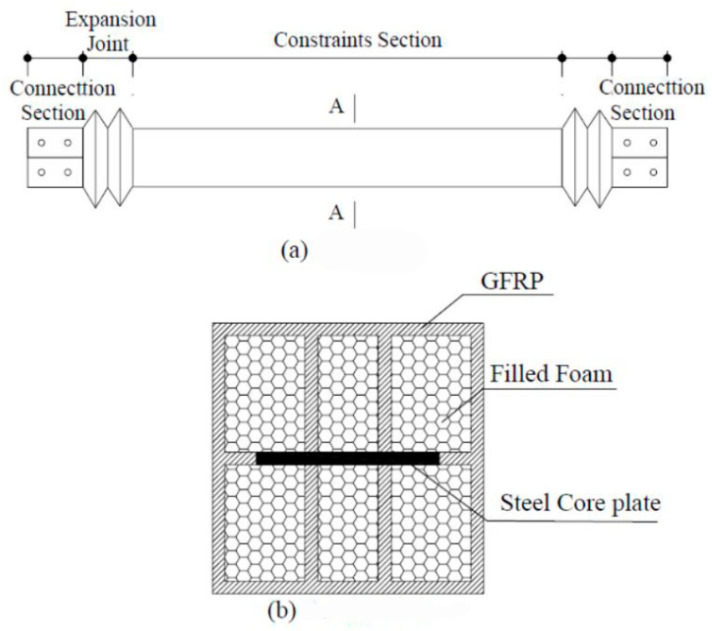
Configuration of the specimen. (**a**) Plan view; (**b**) Section A-A.

The basic structure of the new MFSC-BRB is shown in Figure 1. The stress unit of the inner core of the brace is a one-piece steel plate, with a ribbed GFRP rectangular tube as the restraint unit. The restraining unit GFRP ribs are shaped by introducing resin cured through polyurethane foam strips wrapped with fiberglass cloth. The structural sketch of the ribbed GFRP rectangular tubes is shown in section A-A of Figure 1. A GFRP expansion joint is set at both ends of the new BRB to ensure that the axial force during bracing work is mainly borne by the inner core stress unit. The whole bracing member is formed by one-time integral molding by vacuum infusion, which has the characteristics of sealing and integrity.

The steel core of the new BRB is encapsulated by the integral seal of GFRP. The restraint yield section directly contacts the stress unit of the inner core of the bracing through the rib plate of GFRP and plays a restraining role on the stress unit of the inner core. Both ends of the brace are constructed with retractable expansion joints, which have small axial stiffness and can be designed according to the stiffness of the inner core bearing unit. In order to prevent the new MFSC-BRB from slipping between the restraint unit and inner core stress unit during transportation, installation, and use, a stopper is set in the middle of the inner core stress unit. In order to eliminate the friction between the inner core stress unit and the GFRP restraint unit and to ensure that the inner core stress unit can expand freely while the brace is working, the non-bonded material is coated over the whole length of the area where the inner core stress unit is restrained in the BRB. The new BRB uses silicone as a non-bonded layer material, which has a good delamination effect, bonding force, and deformation ability, and is easy to purchase with low price.

### 2.2. Global Flexural Buckling

The global flexural buckling load *P*_cr,g_ of the BRB specimen, considering the two-end constraint, is calculated using the following equation:

(1) $$P_{cr,g}=\frac{\pi^{2}}{{\left(ul\right)}^{2}}\left(E_{1}I_{1}+E_{2}I_{2}\right)$$

where *E*_1_ and *E*_2_ are the elastic modulus of the inner core and the constraint unit, respectively; *I*_1_ and *I*_2_ are the sectional flexural modulus of the section; *P* is the external load; and *u* is the calculated length coefficient: *u* = 1 when the two ends are hinged, *u* = 0.7 when one end is hinged and the other end is fixed, and *u* = 0.5 when the two ends are fixed.

Usually, the bending stiffness of the inner core unit itself is smaller than that of the constraint element, so the contribution of the inner core unit itself can be ignored. Equation (1) can therefore be simplified as follows:

(2) $$P_{cr,g}=\frac{\pi^{2}E_{2}I_{2}}{{\left(ul\right)}^{2}}\geq F_{y}$$

The concept of the constraining ratio ξ is introduced here. It can be calculated as follows:

(3) $$\xi =\frac{P_{cr,g}}{F_{y}}\geq \frac{\pi^{2}E_{2}I_{2}}{{\left(ul\right)}^{2}F_{y}}$$

Ideally, the steel core reaches full section yielding when ξ≥1. In fact, the strain hardening of steel will lead to the increase of the inner core compressive strength, and the initial imperfection will exist in the brace. The constraining ratio should therefore be enlarged. It is recommended that ξ≥1.5 can be applied in practice.

### 2.3. Gap Control Value

**Figure 2:**
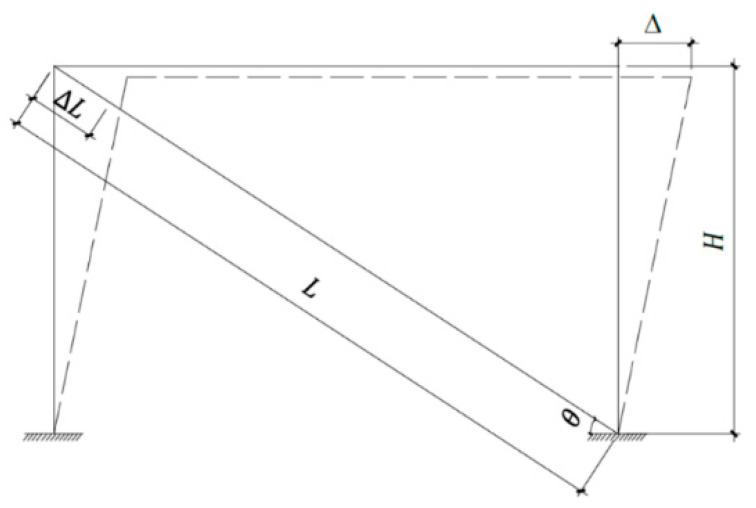
Calculation principle diagram of the brace’s clearance.

As is shown in Figure 2, when the horizontal displacement is Δ, the gap value can be determined as follows:

(4) $${\left(L-\Delta L\right)}^{2}=H^{2}+{\left(L\cos\theta -\Delta \right)}^{2}$$

where L is the axial length of the BRB specimen, ΔL is the axial compression of the BRB specimen, Δ is the horizontal displacement of the structure, θ is the horizontal angle of the BRB specimen, and H is the height of the structure.

According to the Code for the design of high-rising structures (GB 50135-2006) [18], the maximum displacement may be taken as $\Delta =\frac{1}{50}H$, where H is the height of each section of the tower.

When the lateral displacement of 1.5 times occurs, the lateral deformation of the inner core is neglected. Only transverse deformation of the section caused by Poisson’s effect is considered. The gap value δ between the inner core and the constraint unit is shown in Equation (5):

(5) δ=0.015νhsinθcosθ

where ν is the Poisson’s ratio of the steel, $\varepsilon^{\prime }$ is the transverse strain of the inner core, ε is the axial strain of the inner core, Δh is the deformation in the thickness or width direction of the inner core, ΔL is the axial compression of the BRB specimen, and *h* is the cross-section thickness or width of the inner core.

## 3. Design of the MFSC-BRB

**Figure 3:**
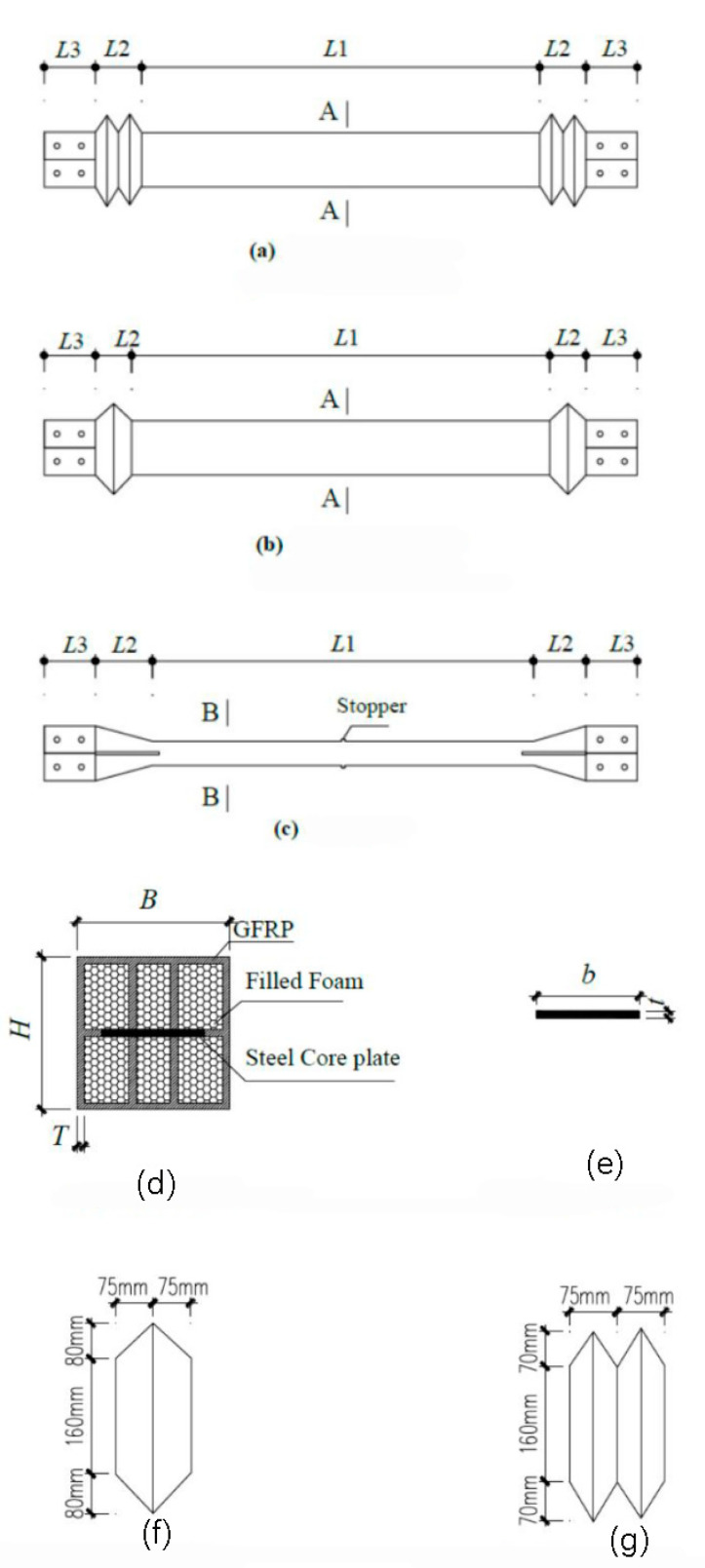
Details of the MFSC-BRB. (**a**) Plan view of BRB (double wave section); (**b**) Plan view of BRB (single wave section); (**c**) Core plate; (**d**) Section A-A; (**e**) Section B-B; (**f**) Single wave section; (**g**) Double wave section.

Taking the length and size of the section of the bottom span diagonal brace of a high-voltage transmission tower as a reference, four specimens were designed by using a 1:3 scale model. The supporting inner core stress unit adopts a slotted steel plate. To prevent relative sliding between the supporting restraint unit and the inner core stress unit, a stopper must be set in the middle of the slotted steel plate. The ribbed GFRP rectangular tube is used as the constraint element, and the constraint yield section is in direct contact with the stress element of the support inner core through the GFRP rib plate, which restricts the stress element of the inner core. GFRP expansion joints are set at both ends of the brace to ensure that the axial force is mainly borne by the inner core stress unit during brace work. The expansion joint can be divided into a single-wave joint and double-wave joint. In this experiment, the deformation capacity and failure mode of the expansion joints with different structural forms were studied. The energy dissipation performances of the braces with different restraint ratios were compared. The structural parameters of the test piece are shown in Table 1 and the design drawing of the test piece is shown in Figure 3.

**Figure 4:**
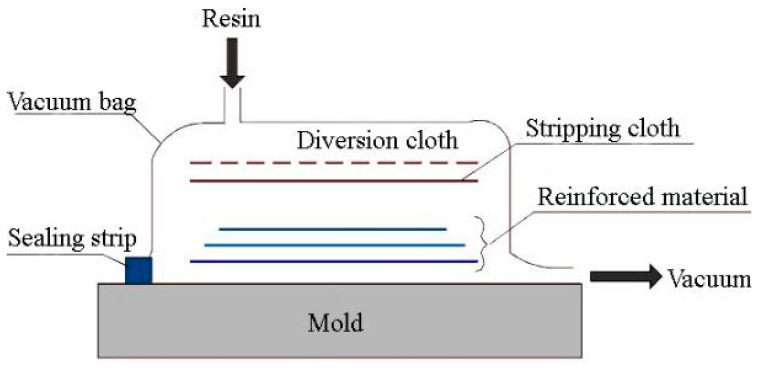
Vacuum-infused molding process.

The MFSC-BRB is manufactured using the vacuum-infused molding process, which has the advantages of flexibility and the capability to integrally form complex composite structural parts all at one time. Compared with the traditional composite molding process, the vacuum-infused molding process has the advantages of strong designability and convenient forming, and can be flexibly designed and mass produced quickly according to needs. It also has the characteristics of sealing and integrity. The basic principle of vacuum-infused molding is shown in Figure 4.

**Figure 5:**
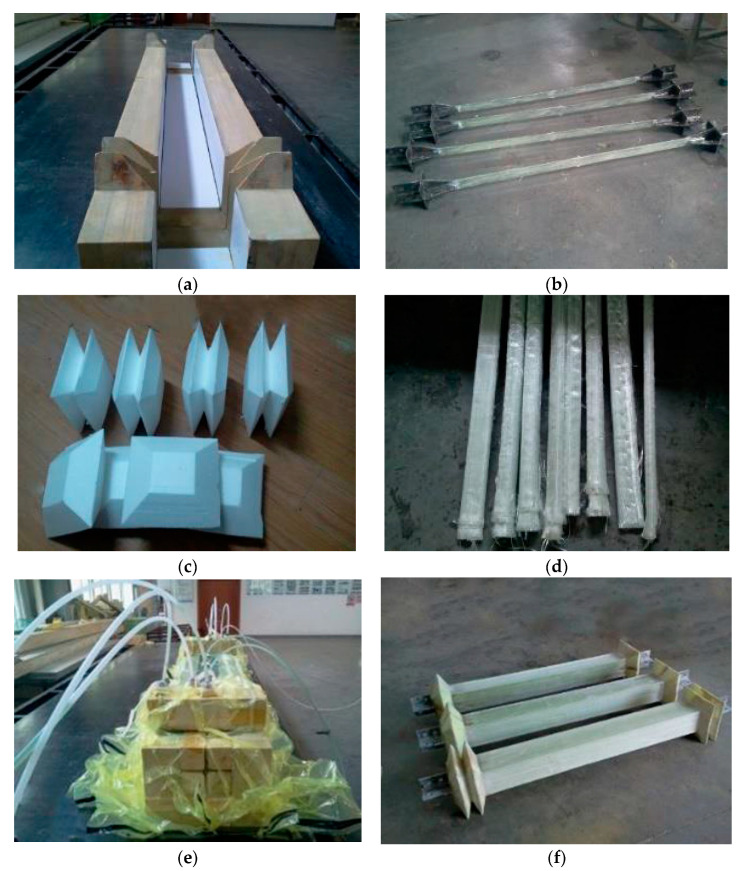
Vacuum-infused molding: (**a**) wood mold; (**b**) spraying the silica gel; (**c**) expansion joint fillers; (**d**) polyurethane foam-wrapped fiber cloth; (**e**) importing the resin; and (**f**) molded specimen.

The process of the vacuum-infused molding is shown in Figure 5. After the steel core is sprayed with silica gel, the foam block is pasted at the two ends of the reserved space for compression. The polyurethane foam tape that outsources the glass fiber cloth and then imports the resin can be molded into GFRP ribs. After the core processing is complete, the glass fiber cloth is laid on the mold, and the steel core and foam core material are put into a wood mold in sequence. The surface glass fiber cloth is then laid on the mold, and the release cloth is laid on the specimen. Then, a vacuum bag is placed to form a sealing system, and the air in the system is removed to form a negative pressure in the mold cavity. The pressure generated by the vacuum presses the resin into the fiber layer through the pre-laid pipe such that the resin can infiltrate the fiber reinforcements, and finally fill the entire mold. After the resin is cured, the products are removed and are subjected to post-treatment. The completed specimen is lightweight and can be easily lifted by a person.

## 4. Experimental Procedure

### 4.1. Experimental Details

**Figure 6:**
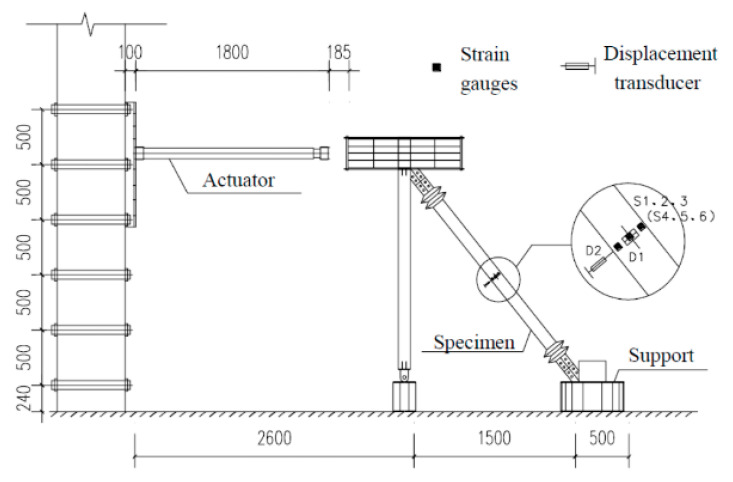
Test device.

A 250 kN electro-hydraulic servo actuator produced by MTS company was used for loading, and the test data were automatically collected by a computer data acquisition system. The test device is shown in Figure 6.

**Figure 7:**
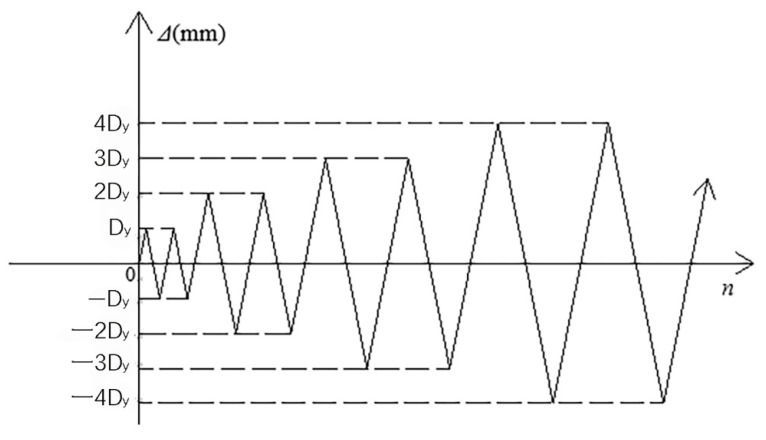
Loading protocol.

Low cycle loading was used in this test. The preloading process was divided into three stages. The first stage was the preloading test. The main purpose of the preloading test was to check whether the instrument worked normally and to ensure that all parts were in good contact. The preload was controlled by the load, with one cycle of 20 kN load applied. Problems found in the preloading test should be adjusted in time. The second stage was the standard loading test. It refers to the requirements of the buckling restrained brace test in relevant regulations. The standard loading scheme calculated by the multiple of yield displacement *D*_y_ was designed to investigate the retardation energy consumption performance of the new MFSC-BRB. The loading protocol used in the experiment is shown in Figure 7. With the increase of *D*_y_, the strain amplitude of each cycle increased from 1 *D*_y_ to 12 *D*_y_. During loading, the displacement of each stage was cycled twice. The third stage was the extra cyclic loading test. The loading displacement of each stage was 2 *D*_y_; the loading cycle was conducted twice for each displacement until the specimen was broken.

### 4.2. Experimental Phenomenon

**Figure 8:**
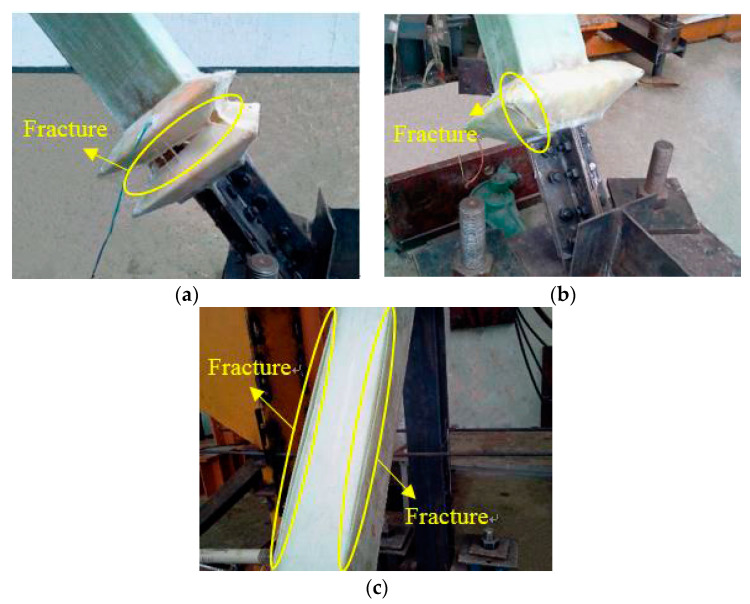
Failure modes: (**a**) tensile fracture of expansion joint of S1; (**b**) tensile fracture of expansion joint of S1; and (**c**) GFRP burst damage of S4.

The failure of the expansion joint and GFRP restrained yield section during test loading was observed as shown in Figure 8. The expansion joint had obvious deformation when the SJ1 axial displacement was loaded back to 28 mm, and the lower expansion joint of the specimen began to crack at the joint. When the SJ1 axial displacement was loaded forward to 40 mm, obvious compression deformation occurred at the lower expansion joint, obvious damage occurred at the corner, and large cracks occurred. Under reverse loading, the lower expansion joint was broken in the middle. When the SJ2 axial displacement was loaded forward to 30 mm, obvious compression deformation could be seen in the upper expansion joint, accompanied by a strong sound. When the SJ2 axial displacement was loaded to 34 mm, the lower expansion joint of the specimen was compressed and cracked. When the SJ3 axial displacement was loaded forward to 30 mm, the top expansion joint begins to show obvious depression deformation. When the SJ3 axial displacement was loaded in the opposite direction to 40 mm, obvious tensile deformation could be seen in the lower expansion joint of the specimen. When the SJ3 axial displacement was loaded forward to 51 mm, the lower expansion joint was compressed obviously and cracks appeared. When the SJ4 axial displacement was positively loaded to 34 mm, some resin peeled off the upper expansion joint. When SJ4 axial displacement was loaded forward to 40 mm, the specimen’s GFRP restraint yield section cracked.

**Figure 9:**
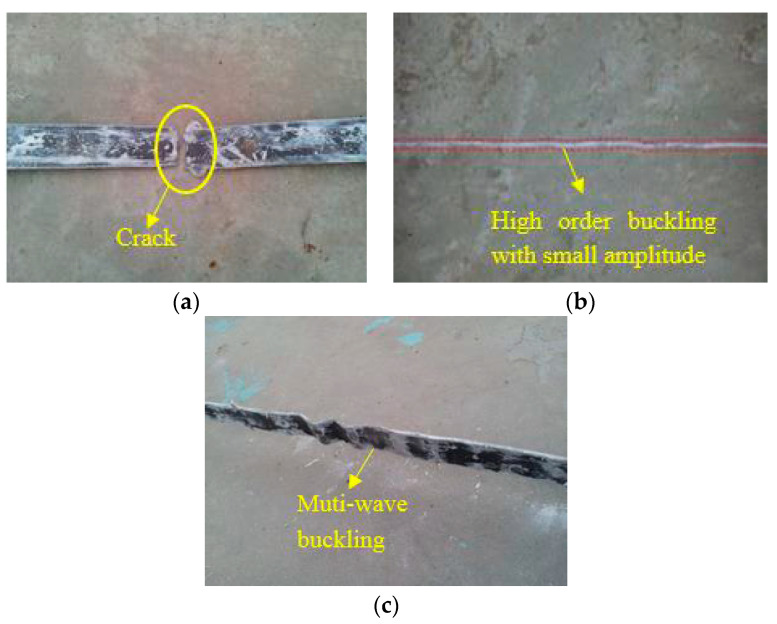
Buckling modal of the inner core: (**a**) damage form of S1’s inner core element, (**b**) buckling modal of S3’s inner core element; and (**c**) buckling modal of S4’s inner core element.

After the test was completed, the specimen was split to check the failure modes of the steel core, as shown in Figure 9. The steel core of SJ1 was broken in the middle part. High-order buckling of the SJ3 steel core occurred with small amplitude. The buckling amplitude of the SJ4 steel core was larger than that of the steel core of SJ3.

The experiments show that the expansion joints of SJ1 were damaged at the corners and troughs, and the expansion joints of SJ2, SJ3, and SJ4 showed good deformation capacity and good integrity in the standard loading stage. When the axial displacement was large, the damage of the expansion joint of the single-wave joint structure was better than that of the double-wave joint structure. The damage of the expansion joint of the double-wave joint structure was concentrated in the angle and groove of the joint, mainly because the stress is concentrated in the angle and groove of the expansion joint, which is prone to damage. Moreover, it has a certain relationship with the manufacturing process of the expansion joint. When the sample is wrapped by fiberglass cloth, the fiberglass cloth at the joint between the fiberglass bottom cloth and the fiberglass cover cloth is discontinuous. When the tension and compression displacement is large, the damage will occur first at this place.

From the buckling form of the core steel of the specimen, it can be judged that the steel core of SJ2 and SJ4 had obvious high-order buckling. During the tests of SJ1, SJ2, SJ3, and SJ4, the energy consumption capacity of the specimens was stable, which indicates that the GFRP restraint mechanism plays a good restraining role.

### 4.3. Comparison of Hysteresis Curves

**Figure 10:**
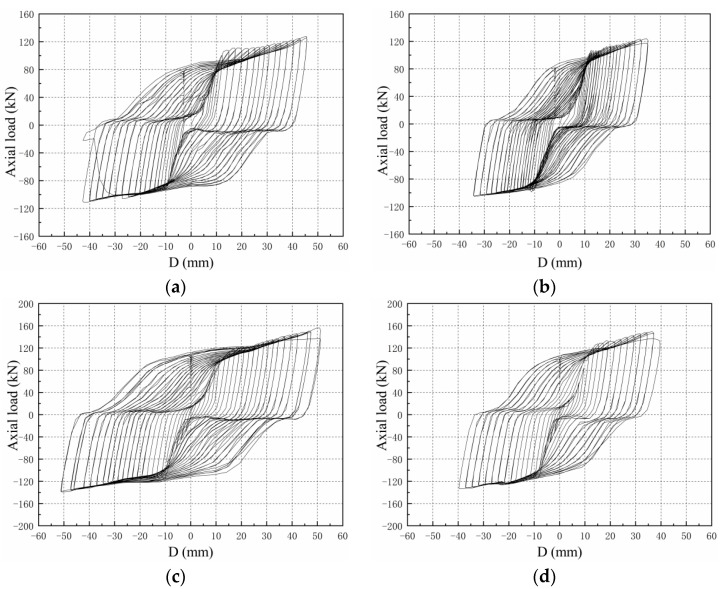
Hysteresis curves of the specimens: (**a**) S1; (**b**) S2; (**c**) S3; and (**d**) S4.

The hysteresis curves of S1–S4 are shown in Figure 10; it can be seen that with the increased loading displacement, the plastic deformation of the steel core gradually increases, the curve appears more and more plump, and the area of the load-displacement curve envelope of the S1–S4 increases with the displacement.

Under the same displacement loading, the compressive load is slightly higher than the tensile load. The main reasons can be concluded as follows: ① First, it is influenced by the transverse expansion of the steel core due to compression, the extrusion of the unbonded silica gel and GFRP, and the friction when relative sliding occurs. ② Second, when the specimen is under pressure, the lateral expansion of the steel core is limited by the GFRP restraint mechanism, so that the steel core is in a three-dimensional stress state, resulting in a slightly higher compressive load than the tensile load under the same level of loading. The ratio of the compressive bearing capacity to the tensile bearing capacity under tension compression displacement at the same level is defined as the compression tension strength ratio. The compression tension strength ratio test results of buckling restrained braces studied abroad are between 1.1 and 1.4. The maximum compression tensile strength ratio of the specimens is 1.23, which is within the range of normal results.

The hysteresis curves show that the lag energy consumption performance of SJ1 is better than that of SJ2, and the lag energy consumption performance of SJ3 is better than that of SJ4, which shows that the greater the restraint ratio of the GFRP constraint, the better the energy consumption capacity of the specimen.

### 4.4. Comparison of Skeleton Curves

**Figure 11:**
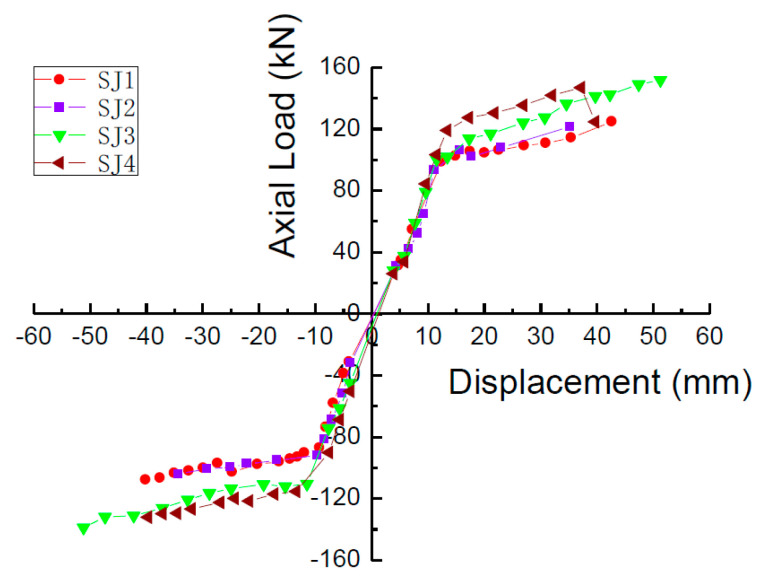
Skeleton curves of the specimens.

The skeleton curves of S1, S2, S3, and S4 are shown in Figure 11. The curves have an obvious yield platform, and also all have the following characteristics: the stiffness of the specimen is large before yielding and the skeleton curve is almost straight. After the specimen yields, the slope of the skeleton curve decreases obviously and exhibits obvious stiffness degradation.

Taking the skew curve turning point of the skeleton curve as the yield point, the measured yield load *F*_y_ and the yield displacement *D*_y_ of each specimen can be obtained. According to the skeleton curve, the maximum load *F*_max_ and the corresponding displacement *D*_max_ can be obtained. The measured yield load *F*_y_, theoretical yield load *F*_yc_, measured yield displacement *D*_y_, maximum load *F*_max_, and corresponding limit displacement *D*_max_ of S1, S2, S3, and S4 are shown in Table 2.

The dissipation coefficient and equivalent viscous damping coefficient are usually used to judge the energy dissipation capacity of the structure.

**Figure 12:**
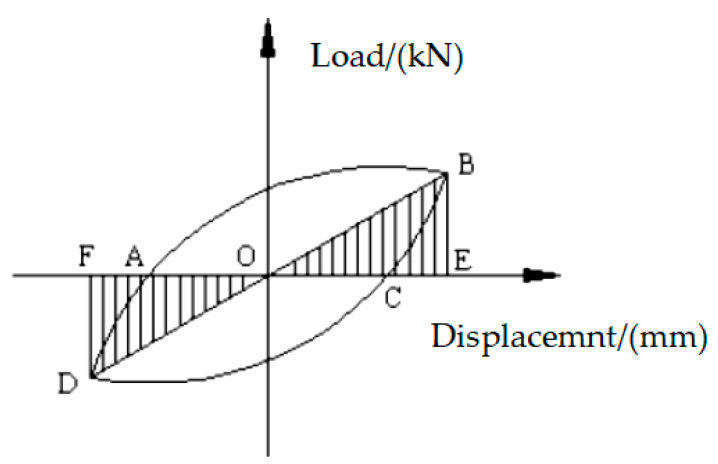
The sketch map of the dissipative ratio.

In the load displacement hysteresis curve, the area surrounded by the hysteresis loop is the energy absorbed and dissipated by the structure. The larger the hysteresis loop area is, the better the energy dissipation capacity of the structure is; the smaller the hysteresis loop area is, the worse the energy dissipation capacity of the structure is. Generally, the energy dissipation coefficient *E* can be used to reflect the energy dissipation capacity of the structure. The calculation formula of the energy dissipation coefficient *E* is shown in Equation (6):

(6) $$E=\frac{S_{\left(ABC+ADC\right)}}{S_{\left(OFD+OBE\right)}}$$

where $S_{\left(ABC+ADC\right)}$ is the area enclosed by the hysteresis loop and $S_{\left(OFD+OBE\right)}$ is the shadowed area in Figure 12.

The equivalent viscous damping coefficient *h*_e_ is also an important index to judge the energy dissipation capacity of the structure. The larger the equivalent viscous damping coefficient is, the stronger the energy dissipation capacity of the structure is. The equivalent viscous damping coefficient *h*_e_ can be determined according to Equation (7):

(7) $$h_{e}=\frac{E}{2\pi }$$

**Figure 13:**
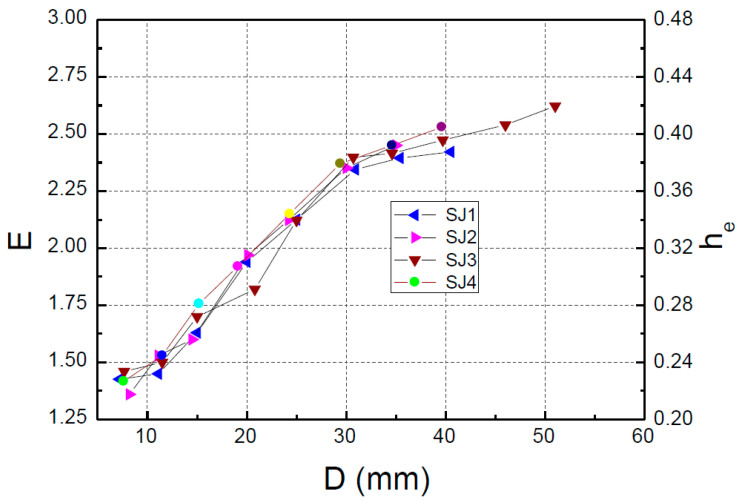
E-D (h_e_-D) curves.

According to the hysteresis curves obtained in the experiment, the relationship between the energy dissipation coefficient *E* and equivalent viscous damping coefficient *h*_e_ with the loading displacement is drawn, as shown in Figure 13. It can be seen from Figure 13 that the energy dissipation capacity *E* and the viscous damping coefficient *h*_e_ gradually increase with increasing loading displacement, and there is no downward trend. The equivalent viscous damping coefficient of the specimen in the energy dissipation stage is much larger than the general damping ratio (0.05) of the structure specified in the Code for Seismic Design of Buildings (GB50011-2010) [19], and the results show that the specimen has a good energy dissipation capacity.

## 5. Conclusions

In this paper, a new type of MFSC-BRB is proposed. The proposed form of MFSC-BRB has the characteristics of good mechanical properties, light weight, and corrosion resistance, and is suitable for high-rise buildings and bridge engineering. The following conclusions can be made:

(1) The MFSC-BRB has the characteristics of a good energy dissipation capacity and stable bearing capacity. During the loading process, the strength of the brace does not decrease significantly, and the envelope area of the hysteresis curve increases with the increase of the displacement.
(2) The equivalent viscous damping coefficient *h*_e_ of the MFSC-BRB increases with the increase of the loading displacement in the standard loading stage, and there is no downward trend; the equivalent viscous damping coefficient *h*_e_ is much larger than the general damping ratio of 0.05 specified in GB50011-2010, which indicates that the MFSC-BRB has a good energy dissipation capacity.
(3) Comparing the deformation capacity and failure modes of the expansion joints of different structures, it was found that when the axial displacement is large, the damage of the expansion joints of the single wave structure is more extensive than that of the double wave structure. Comparing the energy dissipation performance of the brace under different restraint ratios, it was found that the greater the restraint ratio of the GFRP restraint yield section, the better the energy dissipation capacity of the specimen.

## Figures and Tables

**Table 1:** Parameters of the specimens.

Specimen	b×t/mm×mm	$L_{1}/\mathrm{mm}\;$	$L_{2}/\mathrm{mm}$	$L_{3}/\mathrm{mm}$	$\begin{array}{l} B\times H\times T\\ /\mathrm{mm}\times \mathrm{mm}\times \mathrm{mm} \end{array}$	Wave Section
S1	50 × 6	1400	150	150	160 × 160 × 6	Double
S2	50 × 6	1400	150	150	160 × 160 × 4.8	Single
S3	60 × 6	1400	150	150	160 × 160 × 6	Single
S4	60 × 6	1400	150	150	160 × 160 × 4.8	Single

**Table 2:** Characteristics of the envelope curve of the specimens.

Specimen	*F*_y_ (kN)	*F*_yc_ (kN)	*D*_y_ (mm)	*F*_max_ (kN)	*D*_max_ (mm)
S1	92.7	87.9	10	123.7	42
S2	93.5	87.9	10	113.5	34
S3	110.5	105	10	151.8	51
S4	115.4	105	10	147.0	40
